# Obesity and brain structure in schizophrenia – ENIGMA study in 3021 individuals

**DOI:** 10.1038/s41380-022-01616-5

**Published:** 2022-06-14

**Authors:** Sean R. McWhinney, Katharina Brosch, Vince D. Calhoun, Benedicto Crespo-Facorro, Nicolas A. Crossley, Udo Dannlowski, Erin Dickie, Lorielle M. F. Dietze, Gary Donohoe, Stefan Du Plessis, Stefan Ehrlich, Robin Emsley, Petra Furstova, David C. Glahn, Alfonso Gonzalez- Valderrama, Dominik Grotegerd, Laurena Holleran, Tilo T. J. Kircher, Pavel Knytl, Marian Kolenic, Rebekka Lencer, Igor Nenadić, Nils Opel, Julia-Katharina Pfarr, Amanda L. Rodrigue, Kelly Rootes-Murdy, Alex J. Ross, Kang Sim, Antonín Škoch, Filip Spaniel, Frederike Stein, Patrik Švancer, Diana Tordesillas-Gutiérrez, Juan Undurraga, Javier Vázquez-Bourgon, Aristotle Voineskos, Esther Walton, Thomas W. Weickert, Cynthia Shannon Weickert, Paul M. Thompson, Theo G. M. van Erp, Jessica A. Turner, Tomas Hajek

**Affiliations:** 1 Department of Psychiatry, Dalhousie University, Halifax, NS Canada; 2 Department of Psychiatry and Psychotherapy, Philipps-University Marburg, Marburg, Germany; 3 Tri-institutional Center for Translational Research in Neuroimaging and Data Science (TReNDS), Georgia State, Georgia Tech, Emory University, Atlanta, GA USA; 4 Centro de Investigación Biomédica en Red de Salud Mental (CIBERSAM), Madrid, Spain; 5 IBiS, University Hospital Virgen del Rocio, Sevilla, Spain; 6 Department of Psychiatry, School of Medicine, University of Sevilla, Sevilla, Spain; 7 Department of Psychiatry, School of Medicina, Pontificia Universidad Católica de Chile, Santiago, Chile; 8 Department of Psychosis Studies, King’s College London, London, UK; 9 Institute for Translational Psychiatry, University of Münster, Münster, Germany; 10 Centre for Addiction & Mental Health, Department of Psychiatry, University of Toronto, Toronto, ON Canada; 11 Centre for Neuroimaging & Cognitive Genomics (NICOG), Clinical Neuroimaging Laboratory, NCBES Galway Neuroscience Centre, College of Medicine Nursing and Health Sciences, National University of Ireland Galway, Galway, Ireland; 12 Department of Psychiatry, Faculty of Medicine and Health Sciences, Stellenbosch University, Cape Town, South Africa; 13 SAMRC Genomics of Brain Disorders Unit, Cape Town, South Africa; 14 Translational Developmental Neuroscience Section, Division of Psychological and Social Medicine and Developmental Neurosciences, Faculty of Medicine, TU Dresden, Dresden, Germany; 15 National Institute of Mental Health, Klecany, Czech Republic; 16 Department of Psychiatry & Behavioral Sciences, Boston Children’s Hospital, Boston, MA USA; 17 Department of Psychiatry, Harvard Medical School, Boston, MA USA; 18 Olin Neuropsychiatry Research Center, Institute of Living, Hartford, CT USA; 19 School of Medicine, Universidad Finis Terrae, Santiago, Chile; 20 Early Intervention in Psychosis Program, Instituto Psiquiátrico ‘Dr. José Horwitz B.’, Santiago, Chile; 21 Charles University, Third Faculty of Medicine, Prague, Czech Republic; 22 Department of Pscyhiatry and Psychotherapy, University of Lübeck, Lübeck, Germany; 23 Department of Psychiatry, Jena University Hospital/Friedrich-Schiller-University Jena, Jena, Germany; 24 Department of Psychology, Georgia State University, Atlanta, GA USA; 25 West Region, Institute of Mental Health, Singapore, Singapore; 26 Yong Loo Lin School of Medicine, National University of Singapore, Singapore, Singapore; 27 Lee Kong Chian School of Medicine, Nanyang Technological University, Singapore, Singapore; 28 Department of Diagnostic and Interventional Radiology, Institute for Clinical and Experimental Medicine, Prague, Czech Republic; 29 Department of Radiology, Marqués de Valdecilla University Hospital, Valdecilla Biomedical Research Institute IDIVAL, Santander, Spain; 30 Computación Avanzada y Ciencia, Instituto de Física de Cantabria, CSIC, Santander, Spain; 31 Department of Neurology and Psychiatry. Faculty of Medicine, Clínica Alemana Universidad del Desarrollo, Santiago, Chile; 32 Department of Medicine and Psychiatry, School of Medicine, University of Cantabria, Santander, Spain; 33 Department of Psychiatry, Marqués de Valdecilla University Hospital, Valdecilla Biomedical Research Institute IDIVAL, Santander, Spain; 34 Department of Psychology, University of Bath, Bath, UK; 35 Department of Neuroscience and Physiology, SUNY Upstate Medical University, Syracuse, NY USA; 36 Neuroscience Research Australia, Randwick, NSW Australia; 37 School of Psychiatry, University of New South Wales, Sydney, NSW Australia; 38 Imaging Genetics Center, Mark and Mary Stevens Neuroimaging and Informatics Institute, Keck School of Medicine, University of Southern California, Marina del Rey, CA USA; 39 Psychiatry and Human Behavior, University of California Irvine, Irvine, CA USA; 40 Center for the Neurobiology of Learning and Memory, University of California Irvine, Irvine, CA USA

**Keywords:** Schizophrenia, Physiology

## Abstract

Schizophrenia is frequently associated with obesity, which is linked with neurostructural alterations. Yet, we do not understand how the brain correlates of obesity map onto the brain changes in schizophrenia. We obtained MRI-derived brain cortical and subcortical measures and body mass index (BMI) from 1260 individuals with schizophrenia and 1761 controls from 12 independent research sites within the ENIGMA-Schizophrenia Working Group. We jointly modeled the statistical effects of schizophrenia and BMI using mixed effects. BMI was additively associated with structure of many of the same brain regions as schizophrenia, but the cortical and subcortical alterations in schizophrenia were more widespread and pronounced. Both BMI and schizophrenia were primarily associated with changes in cortical thickness, with fewer correlates in surface area. While, BMI was negatively associated with cortical thickness, the significant associations between BMI and surface area or subcortical volumes were positive. Lastly, the brain correlates of obesity were replicated among large studies and closely resembled neurostructural changes in major depressive disorders. We confirmed widespread associations between BMI and brain structure in individuals with schizophrenia. People with both obesity and schizophrenia showed more pronounced brain alterations than people with only one of these conditions. Obesity appears to be a relevant factor which could account for heterogeneity of brain imaging findings and for differences in brain imaging outcomes among people with schizophrenia.

## Introduction

Schizophrenia is among the most disabling and expensive psychiatric disorders [1, 2]. It is frequently associated with brain structural changes, including lower cortical thickness, larger ventricles, and altered subcortical volumes [3, 4]. Individuals with schizophrenia also show greater variability in the structure of individual brain regions than controls [5, 6]. We need to better understand why neurostructural findings vary within the same diagnosis and which factors underlie this heterogeneity. One potential source of differences among individuals with schizophrenia are the comorbidities with medical conditions known to affect the brain [7]. One such condition, which targets the brain and is disproportionately frequent in schizophrenia, is obesity.

Obesity affects 40–60% of patients with psychotic spectrum disorders [8–11], which is significantly more than in the general population [9]. Higher rates of obesity in schizophrenia may be related to shared genetics, pathophysiology, risk factors, including effects of medications or lifestyle factors. Regardless of the ethiology, the presence of obesity has a marked impact on physical health/mortality [12, 13], but could also affect brain structure and related psychiatric outcomes [14, 15]. Indeed, obesity is associated with similar brain imaging alterations in frontal, mesial temporal regions and ventricles, as schizophrenia [16–19]. This overlap has important implications. Obesity could contribute to the varying extent of brain alterations among people with schizophrenia. It could help us identify which individuals with schizophrenia will likely show more pronounced brain changes. This in turn could have clinical implications, as brain alterations are often associated with poor clinical outcomes [20–22]. In addition, perhaps the high rates of obesity, which are shared across major psychiatric disorders, including major depressive disorder (MDD), bipolar disorders (BD) and schizophrenia, could help explain the brain imaging commonalities across these disorders.

Despite the many links between obesity, schizophrenia and the brain, this remains an under researched area. We have previously demonstrated that in people with first episode of psychosis, obesity was associated with advanced brain age and lower cerebellar volume [23, 24] and that BMI was a predictor of future neurostructural alterations [20]. The single previous study in 32 older individuals with schizophrenia found that higher BMI was significantly associated with lower volume of total gray matter, orbitofrontal and prefrontal cortices, and hippocampus [25]. Another study suggested that in schizophrenia, structural brain volume reductions, especially in areas of the reward circuitry, appeared to be related to comorbid metabolic syndrome [26].

We need larger studies in more generalizable samples to better understand how the brain correlates of obesity map onto the brain alterations in schizophrenia. To this goal, we investigated the association between schizophrenia, obesity and neurostructural measures in a large, highly generalizable, multicenter sample from the ENIGMA-Schizophrenia working group. We also compared profiles of obesity-related brain structural alterations with previous large studies in obesity and other neuropsychiatric disorders, including MDD and BD.

## Methods

### Participating sites

The ENIGMA Schizophrenia Working Group brings together researchers with brain imaging and clinical data from people with schizophrenia. Twelve site members of this group contributed individual subject structural MRI data, medication information and body mass index (BMI) values from a total of 1260 individuals with schizophrenia and 1761 healthy controls. Supplementary Tables S1 and S2 list the demographic and clinical details for each cohort. One cohort (COBRE) did not provide BMI for patients, and so only control participants for this site were analyzed. We included this site in analyses to obtain a better estimate of obesity-related brain alterations in controls. The sample is a broad, ecologically valid, and generalizable representation of schizophrenia. All participating sites received approval from local ethics committees, and all participants provided written informed consent.

### Data acquisition and segmentation

High-resolution T1-weighted brain anatomical MRI scans were acquired at each site, see Supplementary Table S3. All groups used the same analytical protocol and performed the same visual and statistical quality assessment, as listed at: http://enigma.ini.usc.edu/protocols/imaging-protocols/. These protocols are standardized across the consortium, are open-source and available online for anyone to scrutinize, to foster open science, replication, and reproducibility. They were applied in large-scale ENIGMA studies of major depression, schizophrenia, ADHD, OCD, PTSD, epilepsy, and autism [27].

Briefly, using the freely available and extensively validated FreeSurfer software, we performed segmentations of 34 cortical regions and 8 subcortical regions, per hemisphere (left and right), based on the Desikan–Killiany atlas. Cortical thickness, cortical surface area, and/or subcortical volume were extracted for each region. We also computed measures of total intracranial volume (ICV) to standardize estimates. Visual quality controls were performed on a region of interest (ROI) level aided by a visual inspection guide including pass/fail segmentation examples. In addition, we generated diagnostic histogram plots for each site and outliers (i.e., ROI volumes), which deviated from the site mean for each structure at 3 or more standard deviations, were flagged for further review. All ROIs failing quality inspection were withheld from subsequent analyses, see Supplementary Tables S4–S6 (on average, 1.33% of data per region). Previous ENIGMA analyses showed that scanner field strength, voxel volume and the version of FreeSurfer used for segmentation did not significantly influence the effect size estimates [28, 29].

### Statistical modeling

In this mega-analysis, we used linear mixed modeling (package *nlme* version 3.1–152 in *R* version 4.1.1) with individual subject cortical thickness, cortical surface area, or subcortical volumes as dependent variables and with both BMI and group (schizophrenia or healthy control) as dependent variables. In each case age, sex, and hemisphere (left or right) were also included as fixed predictors. Total intracranial volume (ICV) was included as a covariate in models of cortical surface area and subcortical volume. Models also included a random effect of hemisphere within participants and a random effect of data collection site. We first tested for additive effects, i.e., whether the association between BMI and brain structure was significant even when controlling for the statistical effect of schizophrenia and other covariates, and would therefore add to the effect of the diagnosis on brain structure. We also checked for interactions and included them where significant.

We created one model per region, with each model including both hemispheres and all of the covariates described above. We used BMI as continuous variable, which captures more variability between participants, increases sensitivity and was the preferred approach in most previous studies [19]. BMI was normally distributed, see Supplementary Fig. S1. We checked the normality of model residuals using QQ plots and tested for multicollinearity using the variance inflation factor (VIF) of all predictor variables included in modeling. Variance in regional volumes was comparable between groups.

We also tested for associations between BMI and available clinical variables, and in any cases where the two were associated we also tested for associations between the particular clinical variable and brain structure. All tests controlled for age, sex, and a random effect of data collection site.

Next, we were interested whether the patterns of changes associated with obesity overlapped with patterns of changes found in major psychiatric disorders. To test this, we ordered the effect sizes of BMI across all regions and using Spearman rank order correlations compared them with rank ordered effect sizes across the same regions, as reported in previous large studies in schizophrenia, MDD or BD. We performed these analyses for measures where the majority of regions showed a significant association with BMI.

We adjusted all *p* values for multiple comparisons using false discovery rate (FDR), with adjusted *p* values reported, at α = 0.05. We calculated effect sizes for between-group differences and associations between BMI and ROI volumes, expressed as standardized coefficients, together with their 95% confidence intervals. The code for all analyses will be provided upon reasonable request.

## Results

### Sample description

We included 3021 participants (1260 individuals with schizophrenia and 1761 healthy controls), see Table 1.

**Table 1:** Demographic, diagnostic and treatment characteristics of sample.

	Controls	Cases	Difference
*N*	1761	1260	
Age, mean (SD)	32.99 (12.05)	32.12 (11.74)	Z = 3.73, *p* < 0.001^a^
BMI, mean (SD)	24.99 (4.97)	25.16 (5.62)	Z = 3.39, *p* < 0.001^a^
Normal weight, *N* (%)	1030 (58.49%)	716 (56.82%)	χ2 = 8.88, *p* = 0.012
Overweight, *N* (%)	486 (27.60%)	322 (25.56%)	
Obese, *N* (%)	245 (13.91%)	222 (17.62%)	
Sex, *N* (%) female	885 (50.26%)	437 (34.68%)	χ2 = 71.75, *p* < 0.001
Treatment at time of scanning, *N* (%)			
*Unmedicated*	–	98 (8.49%)	–
*Atypical antipsychotics*	–	722 (62.51%)	–
*Typical antipsychotics*	–	259 (22.42%)	–
*Atypical and typical antipsychotics*	–	76 (6.58%)	–
Antipsychotic dose, chlorpromazine eq. (mg) mean (SD)	–	454.23 (952.51)	–
Illness duration years, mean (SD)	–	7.01 (11.24)	–
PANSS (Positive), mean (SD)	–	14.39 (32.98)	–
PANSS (Negative), mean (SD)	–	16 (33.15)	–
PANSS (Total), mean (SD)	–	57.58 (37.76)	–
SAPS, mean (SD)	–	20.91 (15.61)	–
SANS, mean (SD)	–	15.26 (13.1)	–

^a^Model coefficients obtained from model controlling for a random effect of site. *PANSS* Positive and Negative Syndrome Scale, *SAPS* Scale for the Assessment of Positive Symptoms, *SANS* Scale for the Assessment of Negative Symptoms

### Regional volume differences by group and BMI

**Fig. 1:**
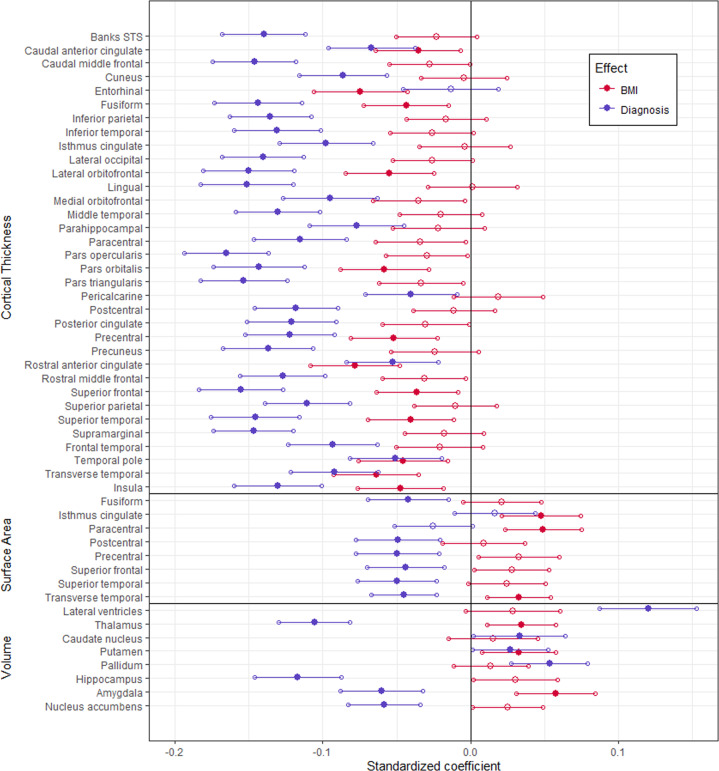
Associations between BMI or diagnosis and brain structure. Standardized coefficients (β) and 95% CI of BMI and diagnosis factor effects on the thickness and surface area of cortical regions, and volume of subcortical regions. Filled markers indicate significant effects (α = 0.05).

When modeled jointly, numerous regions showed significant partial effects of either BMI, diagnosis, or both (Fig. 1). Participants with schizophrenia showed significantly thinner cortex relative to controls in all regions except for the entorhinal cortex. Higher BMI was associated with thinner cortex in many of the same regions as schizophrenia, and it was uniquely associated with thinner entorhinal cortex.

Surface area was significantly lower in participants with schizophrenia relative to controls for a subset of these same regions. Higher BMI was associated with larger surface area in the isthmus of the cingulate gyrus, the paracentral lobule, and transverse temporal cortex.

Lastly, participants with schizophrenia had significantly larger lateral ventricles, caudate nucleus, putamen, and pallidum, but also significantly smaller thalamus, hippocampus, amygdala, and nucleus accumbens, relative to controls. Higher BMI was significantly and additively related to larger volume in a number of these same regions, including thalamus, pallidum, and amygdala.

Overall, all of the regions associated with BMI were also associated with the diagnosis of schizophrenia, with the exception of entorhinal cortex thickness. In contrast, of the 41 unique regions related to schizophrenia, 17 were additively associated with BMI (41.5%). There was no interaction effect of schizophrenia and BMI on cortical thickness, surface area or volume for any brain region (Supplementary Tables S7–S9). The association between BMI and cortical thickness was linear across all regions and all measures, i.e., thickness, surface area or volume, see Supplementary Tables S10–S12 and Supplementary Fig. S2.

### Associations with antipsychotic medications and clinical variables

Among individuals with schizophrenia, the association between BMI and brain structure persisted even when controlling for dose of antipsychotic medications at the time of scanning, see Table 2. Antipsychotic dose at the time of scanning was not significantly related to volume for any subcortical regions, or the thickness or surface area of any cortical regions, see Supplementary Tables S13–S15.

**Table 2:** Partial effects (partial r) of BMI and antipsychotic dose on cortical thickness and subcortical volume, where significant for either (α = 0.05).

			BMI		Antipsychotic dose	
	*Region*	*n*	*r*	*p*	*r*	*p*
Thickness	Banks STS	701	−0.114	0.011	−0.082	0.234
	Caudal middle frontal	731	−0.112	0.011	−0.046	0.341
	Inferior parietal	728	−0.098	0.026	−0.047	0.341
	Lateral occipital	733	−0.124	0.011	−0.039	0.425
	Paracentral	732	−0.110	0.011	−0.085	0.234
	Precentral	692	−0.123	0.011	−0.094	0.234
	Precuneus	731	−0.112	0.011	−0.078	0.234
	Rostral anterior cingulate	728	−0.121	0.011	−0.046	0.341
	Superior frontal	733	−0.098	0.026	−0.056	0.289
	Superior parietal	730	−0.116	0.011	−0.058	0.270
	Transverse temporal	738	−0.122	0.011	−0.064	0.259
Volume	Amygdala	732	0.116	0.015	−0.008	0.834

There was no association between BMI and any other clinical variables, including type of medication (typical vs. atypical (F(1,733) = 0.47, *p* = 0.494)), PANSS negative (F(1,917) = 0.01, *p* = 0.954), positive (F(1,916) = 0.01, *p* = 0.933), or total (F(1,919) = 0.53, *p* = 0.463) scores or with SAPS (F(1,458) = 0.19, *p* = 0.662) or SANS (F(1,772) = 0.15, *p* = 0.701) scores.

### Common effects of BMI and psychiatric illnesses

The rank ordered partial effects of BMI across regions in our sample significantly correlated with the rank ordered effects of BMI across regions as reported by Opel et al. [30] in cortical thickness (ρ = 0.419, *p* = 0.014) and subcortical volume (ρ = 0.905, *p* = 0.005). Also, the partial effect of BMI across regions significantly correlated with patterns of brain changes in MDD; [31] for cortical thickness (ρ = 0.369, *p* = 0.033), but not for subcortical volume (ρ = −0.429, *p* = 0.098).

In contrast, the partial effect of BMI across regions in the present study did not significantly correlate with previously reported group differences in those with BD relative to healthy controls [4, 29] for cortical thickness (ρ = 0.146, *p* = 0.410) or subcortical volume (ρ = −0.310, *p* = 0.462) or in those with schizophrenia relative to healthy controls either in cortical thickness (ρ = 0.056, *p* = 0.755) or subcortical volume (ρ = −0.515, *p* = 0.299) [3, 4].

## Discussion

In this study, BMI was additively associated with structure of many of the same brain regions as schizophrenia, but the cortical and subcortical alterations in schizophrenia were more widespread and pronounced. Both BMI and schizophrenia were primarily associated with changes in cortical thickness, with fewer correlates in surface area. Whereas across all significant regions, BMI was negatively associated with cortical thickness, the significant associations between BMI and surface area or subcortical volumes were positive. The statistical effect of BMI on brain structure was linear in all regions, thus it would be most pronounced in people with obesity, but also manifest in overweight individuals. Lastly, the brain correlates of obesity were replicated against a previous large study [30] and closely resembled neurostructural changes in MDD [31].

The main focus of this study was to investigate how patterns of BMI related brain alterations overlapped with brain changes in schizophrenia. BMI was additively associated with lower cortical thickness in regions which were also thinner in schizophrenia. These included anterior cingulate, temporal pole, and key parts of frontal lobe, that is areas which are considered integral to neuroanatomy of major psychiatric disorders. This is in keeping with previous studies which also demonstrated additive effects of obesity and psychiatric disorders on brain structure [20, 23, 25, 26, 32]. We would expect changes in the regions associated with both conditions to be inflated in studies which did not control for BMI. Consequently, brain alterations in schizophrenia in some of these key regions may be lower than we anticipated. In addition, we would expect these regions to show greater heterogeneity, as they are in part related to the varying presence of obesity. Indeed, some of the regions associated with both BMI and schizophrenia were also the same regions, which showed increased variability in schizophrenia, i.e., putamen and thalamus [5]. Finally, clinicians could expect that individuals with schizophrenia and obesity will show more pronounced brain alterations than people with only one of these conditions. This could be of clinical relevance as more pronounced brain alterations in the above described regions are associated with worse clinical outcomes [21, 22].

Only BMI, but not schizophrenia was associated with thinner entorhinal cortex. Thus, it is possible that findings of thinner entorhinal cortex in schizophrenia from previous studies [33, 34] were related to the uncontrolled presence of obesity. In contrast, schizophrenia was uniquely associated with 23 regions that did not show associations with BMI, see Fig. 1. Thus, previously reported associations between schizophrenia and thinner cortex in these regions should be robust and not confounded by obesity. Importantly, this large study showed no interaction between diagnosis of schizophrenia and BMI in their relationship to brain structure. This is in keeping with other large studies in BD [35] or major depressive disorders [30, 36].

Interestingly, both schizophrenia and BMI were primarily associated with cortical thickness, rather than surface area. Indeed previous large studies also found no [37] or less pronounced/inconsistent [3, 30] associations between obesity or schizophrenia and surface area. Also, these findings fit with studies suggesting that cortical thickness is the more plastic of the two indices and one which is usually associated with external factors [29]. This may also suggest that the observed cortical changes are a consequence of obesity or schizophrenia, rather than representing genetic risk for these conditions. There is a range of mechanisms through which obesity could affect brain structure, including effects of adipokines, oxidative stress, systemic inflammation, insulin resistance/diabetes, hypertension or dyslipidemia [16, 38–43].

We found positive associations between BMI and subcortical volumes, especially in the thalamus, amygdala and putamen. This is consistent with a number of previous studies for amygdala [18, 30, 32, 38, 44, 45], thalamus and putamen [30, 46–50]. Interestingly several studies found the opposite, i.e., associations between obesity and a smaller thalamus or putamen [17, 51], including data from the UK Biobank [18, 44, 52]. These studies typically included older individuals – the average age in our study was 32 years, whereas it was 49 [17], 62 [18] and 77 [51] years in the other studies. It is possible that whereas later in life, the smaller subcortical volumes represent consequences of obesity and related cardiovascular or metabolic issues [51], earlier in life, the larger volumes may represent predispositions for obesity [32, 53]. Indeed, putamen is a part of the brain’s dopaminergic reward system that influences a wide range of motivated behaviors including eating [54], while the amygdala is involved in cue triggered learning and Pavlovian conditioning to hedonic food [55] and in appetitive behavior [56, 57]. Obese individuals show altered neural response towards food-stimuli in regions including amygdala and striatum [58] and this response correlated with BMI [59, 60]. Therefore, the larger volumes of these regions in people with higher BMI may represent risk factors for obesity.

One intriguing question pertains to the role obesity plays in the effects of antipsychotics on brain structure [20, 61]. In our study, BMI remained significantly associated with lower thickness of cortical regions even when we controlled for antipsychotic dose, while the dose of antipsychotics was not significantly associated with brain structure. As the vast majority of individuals were on antipsychotic medications, we could not compare medication naïve or even unmedicated versus medicated individuals. Therefore, we cannot adequately test whether there is an association between antipsychotics and brain structure even when controlling for obesity. However, in our study, the statistical effect of obesity was stronger than that of antipsychotic dose at the time of scanning.

These findings could help explain the marked overlap among major psychiatric disorders in brain imaging alterations [62, 63]. Perhaps this overlap is related to the high rates of obesity, which are present across these disorders. Furthermore, one could expect that the effects of obesity will become relatively more prominent when the effects of the psychiatric illness are relatively smaller. In other words, the impact of obesity on brain changes will be largest in a condition with fewest brain alteration and smallest in conditions which themselves present with pronounced brain changes. In the present study, the patterns of obesity-related alterations resembled brain changes in MDD, which tend to be relatively small and circumscribed, but not those found in BD or schizophrenia, which are usually much more pronounced and diffuse. We could expect that unlike in schizophrenia, controlling for obesity could markedly modify the brain alterations seemingly associated with MDD.

With 3021 individuals, this is the largest study investigating associations between schizophrenia, BMI and brain structure, and one of only 2 such studies [25, 26]. We benefited from the significant methodological refinements and harmonization in the ENIGMA study and from access to a highly generalizable, multi-site sample of individuals with schizophrenia from around the world, thus representing a broad spectrum of individuals with this disorder. Our findings replicate many of the previous findings, which were separately reported in schizophrenia or obesity, but for the first time analyzed these statistical effects jointly. The large sample size allowed us to investigate interactions among variables, which could not be conclusively studied in smaller, less powered studies.

The cross-sectional nature of our study does not allow us to discern the direction of the association, as brain alterations may predate or result from obesity. While we did not have access to other anthropometric or metabolic markers, BMI is the most frequently used measure in similar studies [18, 19, 30, 36, 44] and thus the use of BMI allowed for a more direct comparison with previous results. Dose of medication at the time of testing is a relatively noisy measure, which does not capture the duration of treatment, cumulative exposure, or compliance. Moreover, as our study was not randomized, analyses pertaining to medications could be confounded by individual variability. Cross-diagnostic comparisons would be better addressed by a separate set of analyses in truly cross diagnostic samples, which will be the focus of future ENIGMA studies, pending harmonization of samples.

## Conclusions

To conclude, we confirmed widespread associations between BMI and brain structure. Almost all of the brain regions, which were associated with BMI were also associated with schizophrenia. Therefore, individuals with both obesity and schizophrenia will likely show more pronounced brain alterations than people with only one of these conditions. The regional statistical effects of obesity were most widespread in cortical thickness, with fewer correlates in subcortical volumes or surface area. The BMI related brain alterations closely matched brain changes reported in previous studies of obesity. Interestingly, they also resembled brain alterations previously reported in MDD, but not BD or schizophrenia, suggesting that the impact of obesity may be more pronounced in conditions which themselves present with relatively smaller or more localized changes or are more heterogeneous. In keeping with this, less than half of the regions which were associated with schizophrenia in this study, were also associated with BMI. Obesity appears to be a relevant factor which could account for heterogeneity of brain imaging findings and for differences in brain imaging related psychiatric outcomes among people with schizophrenia, but also other psychiatric disorders. Future studies should investigate whether obesity is a modifiable risk factor for brain alterations in schizophrenia or other psychiatric disorders and whether obesitogenic effects of antipsychotic medications contribute to their associations with brain structure.

## Supplementary information

Supplemental material
