# Correction: Obesity and brain structure in schizophrenia – ENIGMA study in 3021 individuals

**DOI:** 10.1038/s41380-022-01696-3

**Published:** 2022-07-20

**Authors:** Sean R. McWhinney, Katharina Brosch, Vince D. Calhoun, Benedicto Crespo-Facorro, Nicolas A. Crossley, Udo Dannlowski, Erin Dickie, Lorielle M. F. Dietze, Gary Donohoe, Stefan Du Plessis, Stefan Ehrlich, Robin Emsley, Petra Furstova, David C. Glahn, Alfonso Gonzalez- Valderrama, Dominik Grotegerd, Laurena Holleran, Tilo T. J. Kircher, Pavel Knytl, Marian Kolenic, Rebekka Lencer, Igor Nenadić, Nils Opel, Julia-Katharina Pfarr, Amanda L. Rodrigue, Kelly Rootes-Murdy, Alex J. Ross, Kang Sim, Antonín Škoch, Filip Spaniel, Frederike Stein, Patrik Švancer, Diana Tordesillas-Gutiérrez, Juan Undurraga, Javier Vázquez-Bourgon, Aristotle Voineskos, Esther Walton, Thomas W. Weickert, Cynthia Shannon Weickert, Paul M. Thompson, Theo G. M. van Erp, Jessica A. Turner, Tomas Hajek

**Affiliations:** 1 Department of Psychiatry, Dalhousie University, Halifax, NS Canada; 2 Department of Psychiatry and Psychotherapy, Philipps-University Marburg, Marburg, Germany; 3 Tri-institutional Center for Translational Research in Neuroimaging and Data Science (TReNDS), Georgia State, Georgia Tech, Emory University, Atlanta, GA USA; 4 Centro de Investigación Biomédica en Red de Salud Mental (CIBERSAM), Madrid, Spain; 5 IBiS, University Hospital Virgen del Rocio, Sevilla, Spain; 6 Department of Psychiatry, School of Medicine, University of Sevilla, Sevilla, Spain; 7 Department of Psychiatry, School of Medicina, Pontificia Universidad Católica de Chile, Santiago, Chile; 8 Department of Psychosis Studies, King’s College London, London, UK; 9 Institute for Translational Psychiatry, University of Münster, Münster, Germany; 10 Centre for Addiction & Mental Health, Department of Psychiatry, University of Toronto, Toronto, ON Canada; 11 Centre for Neuroimaging & Cognitive Genomics (NICOG), Clinical Neuroimaging Laboratory, NCBES Galway Neuroscience Centre, College of Medicine Nursing and Health Sciences, National University of Ireland Galway, Galway, Ireland; 12 Department of Psychiatry, Faculty of Medicine and Health Sciences, Stellenbosch University, Cape Town, South Africa; 13 SAMRC Genomics of Brain Disorders Unit, Cape Town, South Africa; 14 Translational Developmental Neuroscience Section, Division of Psychological and Social Medicine and Developmental Neurosciences, Faculty of Medicine, TU Dresden, Dresden, Germany; 15 National Institute of Mental Health, Klecany, Czech Republic; 16 Department of Psychiatry & Behavioral Sciences, Boston Children’s Hospital, Boston, MA USA; 17 Department of Psychiatry, Harvard Medical School, Boston, MA USA; 18 Olin Neuropsychiatry Research Center, Institute of Living, Hartford, CT USA; 19 School of Medicine, Universidad Finis Terrae, Santiago, Chile; 20 Early Intervention in Psychosis Program, Instituto Psiquiátrico ‘Dr. José Horwitz B.’, Santiago, Chile; 21 Charles University, Third Faculty of Medicine, Prague, Czech Republic; 22 Department of Pscyhiatry and Psychotherapy, University of Lübeck, Lübeck, Germany; 23 Department of Psychiatry, Jena University Hospital/Friedrich-Schiller-University Jena, Jena, Germany; 24 Department of Psychology, Georgia State University, Atlanta, GA USA; 25 West Region, Institute of Mental Health, Singapore, Singapore; 26 Yong Loo Lin School of Medicine, National University of Singapore, Singapore, Singapore; 27 Lee Kong Chian School of Medicine, Nanyang Technological University, Singapore, Singapore; 28 Department of Diagnostic and Interventional Radiology, Institute for Clinical and Experimental Medicine, Prague, Czech Republic; 29 Department of Radiology, Marqués de Valdecilla University Hospital, Valdecilla Biomedical Research Institute IDIVAL, Santander, Spain; 30 Computación Avanzada y Ciencia, Instituto de Física de Cantabria, CSIC, Santander, Spain; 31 Department of Neurology and Psychiatry. Faculty of Medicine, Clínica Alemana Universidad del Desarrollo, Santiago, Chile; 32 Department of Medicine and Psychiatry, School of Medicine, University of Cantabria, Santander, Spain; 33 Department of Psychiatry, Marqués de Valdecilla University Hospital, Valdecilla Biomedical Research Institute IDIVAL, Santander, Spain; 34 Department of Psychology, University of Bath, Bath, UK; 35 Department of Neuroscience and Physiology, SUNY Upstate Medical University, Syracuse, NY USA; 36 Neuroscience Research Australia, Randwick, NSW Australia; 37 School of Psychiatry, University of New South Wales, Sydney, NSW Australia; 38 Imaging Genetics Center, Mark and Mary Stevens Neuroimaging and Informatics Institute, Keck School of Medicine, University of Southern California, Marina del Rey, CA USA; 39 Psychiatry and Human Behavior, University of California Irvine, Irvine, CA USA; 40 Center for the Neurobiology of Learning and Memory, University of California Irvine, Irvine, CA USA

**Keywords:** Schizophrenia, Physiology

Correction to: *Molecular Psychiatry* 10.1038/s41380-022-01616-5, published online 14 June 2022

The article “Obesity and brain structure in schizophrenia – ENIGMA study in 3021 individuals”, written by Sean R. McWhinney, Katharina Brosch, Vince D. Calhoun, Benedicto Crespo-Facorro, Nicolas A. Crossley, Udo Dannlowski, Erin Dickie, Lorielle M. F. Dietze, Gary Donohoe, Stefan Plessis, Stefan Ehrlich, Robin Emsley, Petra Furstova, David C. Glahn, Alfonso Gonzalez- Valderrama, Dominik Grotegerd, Laurena Holleran, Tilo T. J. Kircher, Pavel Knytl, Marian Kolenic, Rebekka Lencer, Igor Nenadić, Nils Opel, Julia-Katharina Pfarr, Amanda L. Rodrigue, Kelly Rootes-Murdy, Alex J. Ross, Kang Sim, Antonín Škoch, Filip Spaniel, Frederike Stein, Patrik Švancer, Diana Tordesillas-Gutiérrez, Juan Undurraga, Javier Váquez-Bourgon, Aristotle Voineskos, Esther Walton, Thomas W. Weickert, Cynthia Shannon Weickert, Paul M. Thompson, Theo G. M. Erp, Jessica A. Turner, Tomas Hajek, was originally published electronically on the publisher’s internet portal on 14 June 2022 without open access. With the author(s)’ decision to opt for Open Choice the copyright of the article changed on 20 May 2022 to © The Author(s) 2022 and the article is forthwith distributed under a Creative Commons Attribution 4.0 International License, which permits use, sharing, adaptation, distribution and reproduction in any medium or format, as long as you give appropriate credit to the original author(s) and the source, provide a link to the Creative Commons licence, and indicate if changes were made. The images or other third party material in this article are included in the article’s Creative Commons licence, unless indicated otherwise in a credit line to the material. If material is not included in the article’s Creative Commons licence and your intended use is not permitted by statutory regulation or exceeds the permitted use, you will need to obtain permission directly from the copyright holder. To view a copy of this licence, visit http://creativecommons.org/licenses/by/4.0/.

