# Correction: Obesity and brain structure in schizophrenia – ENIGMA study in 3021 individuals

**DOI:** 10.1038/s41380-023-02055-6

**Published:** 2023-04-04

**Authors:** Sean R. McWhinney, Katharina Brosch, Vince D. Calhoun, Benedicto Crespo-Facorro, Nicolas A. Crossley, Udo Dannlowski, Erin Dickie, Lorielle M. F. Dietze, Gary Donohoe, Stefan Du Plessis, Stefan Ehrlich, Robin Emsley, Petra Furstova, David C. Glahn, Alfonso Gonzalez- Valderrama, Dominik Grotegerd, Laurena Holleran, Tilo T. J. Kircher, Pavel Knytl, Marian Kolenic, Rebekka Lencer, Igor Nenadić, Nils Opel, Julia-Katharina Pfarr, Amanda L. Rodrigue, Kelly Rootes-Murdy, Alex J. Ross, Kang Sim, Antonín Škoch, Filip Spaniel, Frederike Stein, Patrik Švancer, Diana Tordesillas-Gutiérrez, Juan Undurraga, Javier Vázquez-Bourgon, Aristotle Voineskos, Esther Walton, Thomas W. Weickert, Cynthia Shannon Weickert, Paul M. Thompson, Theo G. M. van Erp, Jessica A. Turner, Tomas Hajek

**Affiliations:** 1 Department of Psychiatry, Dalhousie University, Halifax, NS Canada; 2 Department of Psychiatry and Psychotherapy, Philipps-University Marburg, Marburg, Germany; 3 Tri-institutional Center for Translational Research in Neuroimaging and Data Science (TReNDS), Georgia State, Georgia Tech, Emory University, Atlanta, GA USA; 4 Centro de Investigación Biomédica en Red de Salud Mental (CIBERSAM), Madrid, Spain; 5 IBiS, University Hospital Virgen del Rocio, Sevilla, Spain; 6 Department of Psychiatry, School of Medicine, University of Sevilla, Sevilla, Spain; 7 Department of Psychiatry, School of Medicina, Pontificia Universidad Católica de Chile, Santiago, Chile; 8 Department of Psychosis Studies, King’s College London, London, UK; 9 Institute for Translational Psychiatry, University of Münster, Münster, Germany; 10 Centre for Addiction & Mental Health, Department of Psychiatry, University of Toronto, Toronto, ON Canada; 11 Centre for Neuroimaging & Cognitive Genomics (NICOG), Clinical Neuroimaging Laboratory, NCBES Galway Neuroscience Centre, College of Medicine Nursing and Health Sciences, National University of Ireland Galway, Galway, Ireland; 12 Department of Psychiatry, Faculty of Medicine and Health Sciences, Stellenbosch University, Cape Town, South Africa; 13 SAMRC Genomics of Brain Disorders Unit, Cape Town, South Africa; 14 Translational Developmental Neuroscience Section, Division of Psychological and Social Medicine and Developmental Neurosciences, Faculty of Medicine, TU Dresden, Dresden, Germany; 15 National Institute of Mental Health, Klecany, Czech Republic; 16 Department of Psychiatry & Behavioral Sciences, Boston Children’s Hospital, Boston, MA USA; 17 Department of Psychiatry, Harvard Medical School, Boston, MA USA; 18 Olin Neuropsychiatry Research Center, Institute of Living, Hartford, CT USA; 19 School of Medicine, Universidad Finis Terrae, Santiago, Chile; 20 Early Intervention in Psychosis Program, Instituto Psiquiátrico ‘Dr. José Horwitz B.’, Santiago, Chile; 21 Charles University, Third Faculty of Medicine, Prague, Czech Republic; 22 Department of Pscyhiatry and Psychotherapy, University of Lübeck, Lübeck, Germany; 23 Department of Psychiatry, Jena University Hospital/Friedrich-Schiller-University Jena, Jena, Germany; 24 Department of Psychology, Georgia State University, Atlanta, GA USA; 25 West Region, Institute of Mental Health, Singapore, Singapore; 26 Yong Loo Lin School of Medicine, National University of Singapore, Singapore, Singapore; 27 Lee Kong Chian School of Medicine, Nanyang Technological University, Singapore, Singapore; 28 Department of Diagnostic and Interventional Radiology, Institute for Clinical and Experimental Medicine, Prague, Czech Republic; 29 Department of Radiology, Marqués de Valdecilla University Hospital, Valdecilla Biomedical Research Institute IDIVAL, Santander, Spain; 30 Computación Avanzada y Ciencia, Instituto de Física de Cantabria, CSIC, Santander, Spain; 31 Department of Neurology and Psychiatry. Faculty of Medicine, Clínica Alemana Universidad del Desarrollo, Santiago, Chile; 32 Department of Medicine and Psychiatry, School of Medicine, University of Cantabria, Santander, Spain; 33 Department of Psychiatry, Marqués de Valdecilla University Hospital, Valdecilla Biomedical Research Institute IDIVAL, Santander, Spain; 34 Department of Psychology, University of Bath, Bath, UK; 35 Department of Neuroscience and Physiology, SUNY Upstate Medical University, Syracuse, NY USA; 36 Neuroscience Research Australia, Randwick, NSW Australia; 37 School of Psychiatry, University of New South Wales, Sydney, NSW Australia; 38 Imaging Genetics Center, Mark and Mary Stevens Neuroimaging and Informatics Institute, Keck School of Medicine, University of Southern California, Marina del Rey, CA USA; 39 Psychiatry and Human Behavior, University of California Irvine, Irvine, CA USA; 40 Center for the Neurobiology of Learning and Memory, University of California Irvine, Irvine, CA USA

**Keywords:** Schizophrenia, Physiology

Correction to: *Molecular Psychiatry* 10.1038/s41380-022-01616-5, published online 14 June 2022

The name of one of the co-authors (Javier Vázquez-Bourgon) had previously been misspelled. This has now been corrected.

The original article has been corrected.

