# Supplementary material for: Obesity and brain structure in schizophrenia – ENIGMA study in 3021 individuals
Source: Mol Psychiatry. 2022 Jun 14;27(9):3731–7. doi: 10.1038/s41380-022-01616-5 (PMC9902274; doi:10.1038/s41380-022-01616-5)
Supplement: Supplementary file 1 — Supplemental material [file 41380_2022_1616_MOESM1_ESM.docx]

**Table S1:** Descriptive statistics of included samples. Abbreviations: PANSS (Positive and Negative Symptoms Scale), SAPS (Scale for the Assessment of Positive Symptoms), SANS (Scale for the Assessment of Negative Symptoms).

|  | **CAMH**  **Toronto, Canada** | | **CASSI**  **Syracuse, USA** | | **COBRE**  **Atlanta, USA** | | **EONCKS**  **Cape Town, South Africa** | | **FOR2107_MR**  **Marburg, Germany** | |
| --- | --- | --- | --- | --- | --- | --- | --- | --- | --- | --- |
|  | Controls | Patients | Controls | Patients | Controls | Patients | Controls | Patients | Controls | Patients |
| N | 83 | 112 | 4 | 47 | 28 | 0 | 88 | 91 | 363 | 35 |
| Age, mean (SD) | 30.24 (9.2) | 43.86 (16.64) | 33.5 (6.24) | 35.51 (8.65) | 40.39 (12.09) | - | 25.34 (7.28) | 24.62 (6.79) | 35.41 (12.74) | 37.63 (11.21) |
| Sex, N (%) female | 38 (45.78%) | 47 (41.96%) | 2 (50%) | 15 (31.91%) | 8 (28.57%) | - | 34 (38.64%) | 24 (26.37%) | 228 (62.81%) | 14 (40%) |
| Obese, N (%) | 10 (12.0%) | 34 (30.4%) | 0 (0.0%) | 26 (55.3%) | 13 (46.4%) | - | 17 (19.3%) | 4 (4.4%) | 32 (8.8%) | 6 (17.1%) |
| Antipsychotic, chlorpromazine eq. (mg) mean (SD) | - | 322.96 (349.36) | - | 589.52 (482.93) | - | - | - | - | - | 404.31 (409.35) |
| Illness Duration months, mean (SD) | - | 9.35 (9.14) | - | 12.17 (6.97) | - | - | - | 0.68 (0.89) | - | 11.81 (7.31) |
| PANSS (Positive), mean (SD) | - | 13.94 (5.85) | - | 7.94 (5.13) | - | - | - | 24.4 (4.56) | - | - |
| PANSS (Negative), mean (SD) | - | 13.82 (5.72) | - | 8.6 (5.86) | - | - | - | 24.52 (6.21) | - | - |
| PANSS (Total), mean (SD) | - | 52.81 (15.56) | - | 32.83 (15.98) | - | - | - | 93.67 (15.32) | - | - |
| SAPS, mean (SD) | - | - | - | - | - | - | - | - | - | 12.03 (11.54) |
| SANS, mean (SD) | - | 26.36 (14.39) | - | - | - | - | - | - | - | 15.53 (11.36) |
|  | **FOR2107_MS**  **Muenster, Germany** | | **Galway**  **Galway, Ireland** | | **IMH**  **Singapore, Singapore** | | **IKEM**  **Prague, Czech Republic** | | **NUDZ**  **Prague, Czech Republic** | |
| N | 217 | 10 | 158 | 48 | 109 | 174 | 79 | 148 | 109 | 226 |
| Age, mean (SD) | 28.55 (10.56) | 34.7 (8.42) | 36.45 (12.59) | 44.23 (10.37) | 33.4 (9.99) | 33.75 (9.49) | 26.8 (5.52) | 29.66 (7.88) | 30.36 (8.25) | 27.16 (7.3) |
| Sex, N (%) female | 142 (65.44%) | 5 (50%) | 65 (41.14%) | 13 (27.08%) | 44 (40.37%) | 57 (32.76%) | 41 (51.9%) | 67 (45.27%) | 67 (61.47%) | 83 (36.73%) |
| Obese, N (%) | 9 (4.1%) | 2 (20.0%) | 17 (10.8%) | 21 (43.8%) | 5 (4.6%) | 16 (9.2%) | 1 (1.3%) | 10 (6.8%) | 7 (6.4%) | 31 (13.7%) |
| Antipsychotic, chlorpromazine eq. (mg) mean (SD) | - | 348.25 (186.16) | - | 2267.58 (3782.53) | - | 202.41 (180.36) | - | 390.81 (224.01) | - | 380.47 (214.73) |
| Illness Duration months, mean (SD) | - | 4.44 (4.82) | - | 17.57 (10.71) | - | 7.4 (8.03) | - | 0.55 (0.66) | - | 0.76 (1.15) |
| PANSS (Positive), mean (SD) | - | - | - | 29.17 (142.98) | - | 10.31 (3.72) | - | 13.91 (4.3) | - | 11.08 (3.49) |
| PANSS (Negative), mean (SD) | - | - | - | 30.25 (142.85) | - | 9.01 (2.98) | - | 16.27 (5.87) | - | 15.81 (5.66) |
| PANSS (Total), mean (SD) | - | - | - | 58.38 (138.9) | - | 39.5 (8.07) | - | 63.94 (15.69) | - | 55.07 (13.73) |
| SAPS, mean (SD) | - | 5.3 (8.91) | - | - | - | - | - | - | - | - |
| SANS, mean (SD) | - | 7.6 (9.38) | - | - | - | - | - | - | - | - |
|  | **MCIC_COBRE**  **Dresden, Germany** | | **OLIN**  **Boston, USA** | | **PAFIP**  **Sevilla, Spain** | | **Santiago**  **Santiago, Chile** | |  |  |
| N | 92 | 106 | 273 | 80 | 102 | 104 | 56 | 79 |  |  |
| Age, mean (SD) | 32.7 (12.09) | 33.55 (11.07) | 39.68 (14.05) | 40.62 (12.92) | 30.2 (7.84) | 29.61 (8.3) | 23.07 (3.45) | 19.95 (3.34) |  |  |
| Sex, N (%) female | 29 (31.52%) | 25 (23.58%) | 129 (47.25%) | 29 (36.25%) | 40 (39.22%) | 43 (41.35%) | 18 (32.14%) | 15 (18.99%) |  |  |
| Obese, N (%) | 15 (16.3%) | 28 (26.4%) | 97 (35.5%) | 38 (47.5%) | 14 (13.7%) | 1 (1.0%) | 5 (8.9%) | 5 (6.3%) |  |  |
| Antipsychotic, chlorpromazine eq. (mg) mean (SD) | - | 534.06 (588.52) | - | - | - | 223.24 (139.99) | - | 548.54 (299.04) |  |  |
| Illness Duration months, mean (SD) | - | 10.42 (10.51) | - | 16.39 (12.66) | - | 0.74 (0.83) | - | 27.17 (21.03) |  |  |
| PANSS (Positive), mean (SD) | - | - | - | - | - | 20.71 (5.98) | - | 16.19 (5.44) |  |  |
| PANSS (Negative), mean (SD) | - | - | - | - | - | 10.29 (3.5) | - | 21.63 (8.45) |  |  |
| PANSS (Total), mean (SD) | - | - | - | - | - | 65.94 (17.34) | - | 71.06 (19.58) |  |  |
| SAPS, mean (SD) | - | 23.28 (16) | - | - | - | 26.05 (13.78) | - | - |  |  |
| SANS, mean (SD) | - | 21.28 (11.72) | - | - | - | 7.1 (10.39) | - | - |  |  |

**Table S2:** Mean and standard deviation BMI for each group, and overall, at each data collection site. Inter-site differences in outcomes based on BMI or other factors were controlled in all models.

|  | BMI (Mean, SD) | | |
| --- | --- | --- | --- |
|  | Overall | Control | Schizophrenia |
| CAMH | 26.46 (6.16) | 24.61 (4.58) | 27.82 (6.82) |
| CASSI | 30.21 (6.64) | 23.22 (3.25) | 30.8 (6.53) |
| COBRE | 30.37 (5.78) | 30.37 (5.78) | n/a |
| EONCKS | 22.72 (5.28) | 23.76 (6.18) | 21.72 (4.03) |
| FOR2107_MR | 24.52 (4.3) | 24.3 (4.16) | 26.82 (5.06) |
| FOR2107_MS | 23.29 (3.81) | 23.11 (3.62) | 27.12 (5.74) |
| Galway | 25.78 (4.62) | 24.58 (3.93) | 29.75 (4.52) |
| IMH | 23.14 (4.14) | 23.63 (3.5) | 22.83 (4.48) |
| IKEM | 23.45 (3.81) | 23.15 (3.13) | 23.62 (4.13) |
| NUDZ | 24.4 (4.33) | 23.59 (3.76) | 24.79 (4.54) |
| MCIC_COBRE | 26.68 (5.34) | 25.75 (4.95) | 27.48 (5.56) |
| OLIN | 29.15 (6.38) | 28.82 (6.32) | 30.27 (6.51) |
| PAFIP | 23.89 (4.07) | 25.69 (4.2) | 22.14 (3.07) |
| Santiago | 23.76 (3.93) | 24.77 (3.82) | 23.05 (3.87) |

**Table S3:** Image acquisition parameters and software versions used at each site

| Site | FreeSurfer | MRI | Imaging protocols | Orientation |
| --- | --- | --- | --- | --- |
| CAMH | 5.3 | GE 1.5T | SPGR, TR/TE/TI=12.3/5.3/300ms, flip angle=20°, 256x256x128 matrix, FOV=240x240mm, slice thickness=1.5mm | Axial |
| COBRE | 5.3 | 3T Siemens TIM Trio | T1-weighted images were acquired with  a 5-echo multi-echo MPRAGE sequence  [TE (echo times) = 1.64, 3.5, 5.36, 7.22,  9.08 ms, TR (repetition time) = 2.53 s, TI (inversion time) = 1.2 s, 7○ flip angle, number of excitations (NEX) = 1, slice thickness = 1 mm, FOV (field of view) = 256 mm, resolution = 256x256] | Sagittal |
| CASSI | 5.1.0 | 3T Phillips Achieva | T1 weighted high-resolution anatomical scans, MPRAGE, 1mm slice thickness, no gap, 180 slices, TR = 5.4ms, TE = 2.4ms, field of view 256mm | Sagittal |
| EONCKS | 6.0 | 3T Siements Allegra | 3D T1-weighted MPRAGE; TE=4.88ms, TR=2080ms; 0.9x0.9x1.0 mm voxel size with 176 slices, FOV (field of view)=230 mm | Sagittal |
| FOR2107_MR | 5.3 | 3T Siemens Magnetom Trio | 3D T1-weighted MPRAGE; TE=2.26 ms, TR=1900ms, TI=900ms; 1x1x1 mm voxel size with 176 slices and 0.5 mm slice gap, 9° flip angle | Sagittal |
| FOR2107_MS | 5.3 | 3T Siemens PRISMA | 3D T1-weighted MPRAGE; TE=2.28 ms, TR=2130ms, TI=900ms; 1x1x1 mm voxel size with 192 slices, 8° flip angle | Sagittal |
| Galway | 5.3 | 3T Philips Achieva | 3D T1-weighted MPRAGE; TE=2.28 ms, TR=2130ms, TI=900ms; 1x1x1 mm voxel size with 192 slices, 8° flip angle | Sagittal |
| Halifax | 5.3 | 1.5T GE Signa | 3D T1-weighted SPGR; TE=5ms, TR=25ms, TI=0ms; .9375 x .9375 x 1.5 mm voxel size with 125 slices, 40° flip angle | Coronal |
| IMH | 5.3 | Philips Achieva | T1 weighted MPRAGE, 180 axial slices of 0.9mm thickness with no gap, FOV = 230x230 mm2, matrix 256x204, voxel size =0.89x0.89x0.9 mm3, TR=7.2 s, TE=3.3 ms, FA=8° | Axial |
| IKEM | 6 | 3T Siemens Trio MRI scanner | T1-weighted 3D MPRAGE scans (TE = 4.63 ms, TR = 2300 ms, bandwidth 130 Hz/pixel, FOV = 256 × 256 mm, matrix 256 × 256, voxel size 1 × 1 × 1 mm3) equipped with standard head coil. | Sagittal |
| NUDZ | 6 | 3T Siemens Prisma MAGNETOM | T1-weighted 3D MPRAGE scans (TE = 4.63 ms, TR = 2300 ms, bandwidth 130 Hz/pixel, FOV = 256 × 256 mm, matrix 256 × 256, voxel size 1 × 1 × 1 mm3) equipped with standard head coil. | Sagittal |
| MCIC_COBRE | 4.0.1 | 1.5T Siemens, 3T GE | TR = 2530 ms for 3 T, TR = 12 ms for 1.5 T; TE = 3.79 ms for 3 T, TE = 4.76 ms for 1.5 T; FA = 7 for 3 T, FA = 20 for 1.5 T; TI = 1100 for 3 T; Bandwidth = 181 for 3 T, Bandwidth = 110 for 1.5 T; 0.625×0.625 mm voxel size; slice thickness 1.5 mm; FOV 256×256×128 cm matrix; FOV = 16 cm (could be increased to 18 cm when needed for full brain coverage). | Coronal |
| OLIN | 5.1 | 3T Alegra | T1-weighted, 3D magnetizationprepared rapid gradient-echo(MPRAGE) sequence(TR/TE/TI=2200/4.13/766 ms, flip angle=13°, voxel size [isotropic]=0.8mm, image size=240 x 320 x 208 voxels), with axial slices parallel to the AC-PC line. | Axial |
| PAFIP | 5 | GE 1.5T | Three-dimensional T1-weighted images, using a spoiled grass (SPGR) sequence acquired in the coronal plane with: echo time (TE)=5 ms, repetition time (TR)=24 ms, numbers of excitations  (NEX)=2, rotation angle=45°, field of view (FOV)=26×19.5 cm, slice thickness=1.5mm and a matrix of 256×192. | Coronal |
| Santiago | 6 | Phillips 3T Ingenia | 3D T1 TFE; voxel size 1.0mm3 isotropic; min. TI delay 965.2 / TE 3.5 / TR 7.7/ Flip angle 8°. | Sagittal |

**Table S4:** Number of participants removed from analysis in each region based on poor data quality or unreliable segmentation (cortical thickness)

|  | Removed - n (%) | | |
| --- | --- | --- | --- |
|  | Overall | Control | Schizophrenia |
| Banks STS | 204 (3.38%) | 103 (2.92%) | 101 (4.01%) |
| Caudal anterior cingulate | 70 (1.16%) | 26 (0.74%) | 44 (1.75%) |
| Caudal middle frontal | 42 (0.7%) | 19 (0.54%) | 23 (0.91%) |
| Cuneus | 77 (1.27%) | 34 (0.97%) | 43 (1.71%) |
| Entorhinal | 195 (3.23%) | 103 (2.92%) | 92 (3.65%) |
| Fusiform | 49 (0.81%) | 22 (0.62%) | 27 (1.07%) |
| Inferior parietal | 57 (0.94%) | 29 (0.82%) | 28 (1.11%) |
| Inferior temporal | 90 (1.49%) | 39 (1.11%) | 51 (2.02%) |
| Isthmus cingulate | 47 (0.78%) | 15 (0.43%) | 32 (1.27%) |
| Lateral occipital | 39 (0.65%) | 15 (0.43%) | 24 (0.95%) |
| Lateral orbitofrontal | 46 (0.76%) | 19 (0.54%) | 27 (1.07%) |
| Lingual | 56 (0.93%) | 30 (0.85%) | 26 (1.03%) |
| Medial orbitofrontal | 62 (1.03%) | 27 (0.77%) | 35 (1.39%) |
| Middle temporal | 103 (1.7%) | 46 (1.31%) | 57 (2.26%) |
| Parahippocampal | 25 (0.41%) | 12 (0.34%) | 13 (0.52%) |
| Paracentral | 54 (0.89%) | 28 (0.8%) | 26 (1.03%) |
| Pars opercularis | 31 (0.51%) | 12 (0.34%) | 19 (0.75%) |
| Pars orbitalis | 40 (0.66%) | 22 (0.62%) | 18 (0.71%) |
| Pars triangularis | 42 (0.7%) | 19 (0.54%) | 23 (0.91%) |
| Pericalcarine | 144 (2.38%) | 78 (2.21%) | 66 (2.62%) |
| Postcentral | 189 (3.13%) | 129 (3.66%) | 60 (2.38%) |
| Posterior cingulate | 49 (0.81%) | 28 (0.8%) | 21 (0.83%) |
| Precentral | 192 (3.18%) | 135 (3.83%) | 57 (2.26%) |
| Precuneus | 49 (0.81%) | 23 (0.65%) | 26 (1.03%) |
| Rostral anterior cingulate | 78 (1.29%) | 32 (0.91%) | 46 (1.83%) |
| Rostral middle frontal | 52 (0.86%) | 30 (0.85%) | 22 (0.87%) |
| Superior frontal | 53 (0.88%) | 34 (0.97%) | 19 (0.75%) |
| Superior parietal | 38 (0.63%) | 20 (0.57%) | 18 (0.71%) |
| Superior temporal | 157 (2.6%) | 73 (2.07%) | 84 (3.33%) |
| Supramarginal | 103 (1.7%) | 50 (1.42%) | 53 (2.1%) |
| Frontal temporal | 48 (0.79%) | 27 (0.77%) | 21 (0.83%) |
| Temporal pole | 57 (0.94%) | 36 (1.02%) | 21 (0.83%) |
| Transverse temporal | 27 (0.45%) | 14 (0.4%) | 13 (0.52%) |
| Insula | 148 (2.45%) | 81 (2.3%) | 67 (2.66%) |

**Table S5:** Number of participants removed from analysis in each region based on poor data quality or unreliable segmentation (cortical surface area)

|  | Removed - n (%) | | |
| --- | --- | --- | --- |
|  | Overall | Control | Schizophrenia |
| Banks STS | 218 (3.61%) | 116 (3.29%) | 102 (4.05%) |
| Caudal anterior cingulate | 73 (1.21%) | 38 (1.08%) | 35 (1.39%) |
| Caudal middle frontal | 52 (0.86%) | 29 (0.82%) | 23 (0.91%) |
| Cuneus | 91 (1.51%) | 46 (1.31%) | 45 (1.79%) |
| Entorhinal | 205 (3.39%) | 99 (2.81%) | 106 (4.21%) |
| Fusiform | 49 (0.81%) | 20 (0.57%) | 29 (1.15%) |
| Inferior parietal | 63 (1.04%) | 32 (0.91%) | 31 (1.23%) |
| Inferior temporal | 90 (1.49%) | 42 (1.19%) | 48 (1.9%) |
| Isthmus cingulate | 50 (0.83%) | 18 (0.51%) | 32 (1.27%) |
| Lateral occipital | 51 (0.84%) | 28 (0.8%) | 23 (0.91%) |
| Lateral orbitofrontal | 41 (0.68%) | 17 (0.48%) | 24 (0.95%) |
| Lingual | 51 (0.84%) | 29 (0.82%) | 22 (0.87%) |
| Medial orbitofrontal | 60 (0.99%) | 32 (0.91%) | 28 (1.11%) |
| Middle temporal | 102 (1.69%) | 50 (1.42%) | 52 (2.06%) |
| Parahippocampal | 59 (0.98%) | 31 (0.88%) | 28 (1.11%) |
| Paracentral | 55 (0.91%) | 28 (0.8%) | 27 (1.07%) |
| Pars opercularis | 64 (1.06%) | 31 (0.88%) | 33 (1.31%) |
| Pars orbitalis | 44 (0.73%) | 19 (0.54%) | 25 (0.99%) |
| Pars triangularis | 64 (1.06%) | 36 (1.02%) | 28 (1.11%) |
| Pericalcarine | 142 (2.35%) | 80 (2.27%) | 62 (2.46%) |
| Postcentral | 203 (3.36%) | 134 (3.8%) | 69 (2.74%) |
| Posterior cingulate | 44 (0.73%) | 29 (0.82%) | 15 (0.6%) |
| Precentral | 188 (3.11%) | 128 (3.63%) | 60 (2.38%) |
| Precuneus | 44 (0.73%) | 19 (0.54%) | 25 (0.99%) |
| Rostral anterior cingulate | 63 (1.04%) | 30 (0.85%) | 33 (1.31%) |
| Rostral middle frontal | 52 (0.86%) | 28 (0.8%) | 24 (0.95%) |
| Superior frontal | 55 (0.91%) | 33 (0.94%) | 22 (0.87%) |
| Superior parietal | 53 (0.88%) | 28 (0.8%) | 25 (0.99%) |
| Superior temporal | 177 (2.93%) | 86 (2.44%) | 91 (3.61%) |
| Supramarginal | 116 (1.92%) | 56 (1.59%) | 60 (2.38%) |
| Frontal temporal | 25 (0.41%) | 13 (0.37%) | 12 (0.48%) |
| Temporal pole | 49 (0.81%) | 31 (0.88%) | 18 (0.71%) |
| Transverse temporal | 54 (0.89%) | 37 (1.05%) | 17 (0.67%) |
| Insula | 144 (2.38%) | 77 (2.19%) | 67 (2.66%) |

**Table S6:** Number of participants removed from analysis in each region based on poor data quality or unreliable segmentation (cortical surface area)

|  | Removed - n (%) | | |
| --- | --- | --- | --- |
|  | Overall | Control | Schizophrenia |
| Lateral ventricles | 95 (1.57%) | 59 (1.68%) | 36 (1.43%) |
| Thalamus | 24 (0.4%) | 17 (0.48%) | 7 (0.28%) |
| Caudate nucleus | 32 (0.53%) | 16 (0.45%) | 16 (0.63%) |
| Putamen | 83 (1.37%) | 37 (1.05%) | 46 (1.83%) |
| Pallidum | 137 (2.27%) | 63 (1.79%) | 74 (2.94%) |
| Hippocampus | 35 (0.58%) | 19 (0.54%) | 16 (0.63%) |
| Amygdala | 48 (0.79%) | 29 (0.82%) | 19 (0.75%) |
| Nucleus accumbens | 53 (0.88%) | 27 (0.77%) | 26 (1.03%) |

**Table S7** Interactions between diagnosis effect and BMI in predicting cortical thickness for each region, using FDR-adjusted p-values

| Region | Estimate | Std. Error | DF | t | p (Raw) | p (FDR) |
| --- | --- | --- | --- | --- | --- | --- |
| Banks STS | 0.00 | 0.00 | 2859 | -0.25 | 0.806 | 0.904 |
| Caudal anterior cingulate | 0.00 | 0.00 | 2977 | 1.43 | 0.154 | 0.717 |
| Caudal middle frontal | 0.00 | 0.00 | 3007 | 1.19 | 0.236 | 0.717 |
| Cuneus | 0.00 | 0.00 | 2977 | 0.68 | 0.498 | 0.727 |
| Entorhinal | 0.00 | 0.00 | 2888 | -0.15 | 0.877 | 0.904 |
| Fusiform | 0.00 | 0.00 | 2999 | 1.01 | 0.315 | 0.717 |
| Inferior parietal | 0.00 | 0.00 | 2989 | 0.39 | 0.693 | 0.904 |
| Inferior temporal | 0.00 | 0.00 | 2963 | 0.92 | 0.360 | 0.717 |
| Isthmus cingulate | 0.00 | 0.00 | 3001 | -0.83 | 0.409 | 0.717 |
| Lateral occipital | 0.00 | 0.00 | 3009 | -0.19 | 0.847 | 0.904 |
| Lateral orbitofrontal | 0.00 | 0.00 | 3004 | 1.98 | 0.048 | 0.408 |
| Lingual | 0.00 | 0.00 | 2992 | 0.85 | 0.398 | 0.717 |
| Medial orbitofrontal | 0.00 | 0.00 | 2989 | 0.83 | 0.409 | 0.717 |
| Middle temporal | 0.00 | 0.00 | 2945 | 0.65 | 0.513 | 0.727 |
| Parahippocampal | 0.00 | 0.00 | 3019 | 0.71 | 0.476 | 0.727 |
| Paracentral | 0.00 | 0.00 | 2998 | 0.88 | 0.378 | 0.717 |
| Pars opercularis | 0.00 | 0.00 | 3015 | 2.38 | 0.018 | 0.306 |
| Pars orbitalis | 0.00 | 0.00 | 3005 | 2.49 | 0.013 | 0.306 |
| Pars triangularis | 0.00 | 0.00 | 3005 | 0.98 | 0.328 | 0.717 |
| Pericalcarine | 0.00 | 0.00 | 2922 | -0.80 | 0.422 | 0.717 |
| Postcentral | 0.00 | 0.00 | 2921 | 0.02 | 0.981 | 0.981 |
| Posterior cingulate | 0.00 | 0.00 | 2999 | 0.70 | 0.482 | 0.727 |
| Precentral | 0.00 | 0.00 | 2924 | 2.13 | 0.033 | 0.374 |
| Precuneus | 0.00 | 0.00 | 3004 | 0.22 | 0.824 | 0.904 |
| Rostral anterior cingulate | 0.00 | 0.00 | 2974 | 0.17 | 0.862 | 0.904 |
| Rostral middle frontal | 0.00 | 0.00 | 3000 | 1.25 | 0.211 | 0.717 |
| Superior frontal | 0.00 | 0.00 | 3004 | 0.32 | 0.746 | 0.904 |
| Superior parietal | 0.00 | 0.00 | 3012 | 0.32 | 0.746 | 0.904 |
| Superior temporal | 0.00 | 0.00 | 2910 | 0.92 | 0.358 | 0.717 |
| Supramarginal | 0.00 | 0.00 | 2953 | 1.17 | 0.243 | 0.717 |
| Frontal temporal | 0.00 | 0.00 | 2997 | 0.94 | 0.349 | 0.717 |
| Temporal pole | 0.00 | 0.00 | 2994 | 0.45 | 0.651 | 0.885 |
| Transverse temporal | 0.00 | 0.00 | 3018 | 1.39 | 0.164 | 0.717 |
| Insula | 0.00 | 0.00 | 2912 | 1.55 | 0.122 | 0.717 |

**Table S8** Interactions between diagnosis effect and BMI in predicting cortical surface area for each region, using FDR-adjusted p-values

| Region | Estimate | Std. Error | DF | t | p (Raw) | p (FDR) |
| --- | --- | --- | --- | --- | --- | --- |
| Banks STS | 1.48 | 0.76 | 2844 | 1.95 | 0.051 | 0.394 |
| Caudal anterior cingulate | 0.78 | 0.70 | 2970 | 1.11 | 0.268 | 0.651 |
| Caudal middle frontal | -1.83 | 1.95 | 2996 | -0.94 | 0.348 | 0.776 |
| Cuneus | 0.67 | 1.21 | 2962 | 0.55 | 0.582 | 0.781 |
| Entorhinal | -0.25 | 0.47 | 2878 | -0.53 | 0.597 | 0.781 |
| Fusiform | 1.50 | 1.95 | 2994 | 0.77 | 0.442 | 0.776 |
| Inferior parietal | 4.24 | 3.49 | 2983 | 1.22 | 0.224 | 0.635 |
| Inferior temporal | 2.69 | 2.38 | 2960 | 1.13 | 0.257 | 0.651 |
| Isthmus cingulate | -0.05 | 0.80 | 2996 | -0.06 | 0.950 | 0.975 |
| Lateral occipital | 1.52 | 3.27 | 2997 | 0.47 | 0.642 | 0.792 |
| Lateral orbitofrontal | -0.90 | 1.57 | 3004 | -0.58 | 0.564 | 0.781 |
| Lingual | 4.58 | 2.41 | 2998 | 1.90 | 0.058 | 0.394 |
| Medial orbitofrontal | -0.15 | 1.12 | 2992 | -0.13 | 0.893 | 0.949 |
| Middle temporal | 3.14 | 2.19 | 2946 | 1.44 | 0.151 | 0.498 |
| Parahippocampal | 0.01 | 0.47 | 2982 | 0.03 | 0.975 | 0.975 |
| Paracentral | -2.86 | 0.96 | 2993 | -2.97 | 0.003 | 0.102 |
| Pars opercularis | -0.86 | 1.25 | 2981 | -0.69 | 0.491 | 0.776 |
| Pars orbitalis | 0.72 | 0.50 | 3001 | 1.43 | 0.153 | 0.498 |
| Pars triangularis | -0.79 | 1.18 | 2986 | -0.67 | 0.502 | 0.776 |
| Pericalcarine | 0.71 | 1.57 | 2923 | 0.45 | 0.652 | 0.792 |
| Postcentral | -2.07 | 2.40 | 2911 | -0.86 | 0.388 | 0.776 |
| Posterior cingulate | -0.31 | 0.90 | 3000 | -0.35 | 0.725 | 0.850 |
| Precentral | -3.78 | 2.65 | 2923 | -1.43 | 0.153 | 0.498 |
| Precuneus | -1.49 | 2.52 | 3005 | -0.59 | 0.555 | 0.781 |
| Rostral anterior cingulate | 1.46 | 0.72 | 2984 | 2.01 | 0.044 | 0.394 |
| Rostral middle frontal | 0.71 | 3.93 | 2996 | 0.18 | 0.857 | 0.940 |
| Superior frontal | -6.37 | 4.22 | 3000 | -1.51 | 0.132 | 0.498 |
| Superior parietal | -0.84 | 3.47 | 2995 | -0.24 | 0.809 | 0.917 |
| Superior temporal | -3.01 | 2.15 | 2890 | -1.40 | 0.161 | 0.498 |
| Supramarginal | -2.30 | 2.89 | 2940 | -0.80 | 0.426 | 0.776 |
| Frontal temporal | 0.13 | 0.19 | 3019 | 0.69 | 0.488 | 0.776 |
| Temporal pole | 0.27 | 0.32 | 2997 | 0.83 | 0.406 | 0.776 |
| Transverse temporal | -0.52 | 0.34 | 2990 | -1.56 | 0.119 | 0.498 |
| Insula | -2.97 | 1.33 | 2907 | -2.23 | 0.026 | 0.394 |

**Table S9** Interactions between diagnosis effect and BMI in predicting subcortical volume for each region, using FDR-adjusted p-values

| Region | Estimate | Std. Error | DF | t | p (Raw) | p (FDR) |
| --- | --- | --- | --- | --- | --- | --- |
| Lateral ventricles | -12.98 | 19.18 | 2933 | -0.68 | 0.499 | 0.979 |
| Thalamus | 0.10 | 3.99 | 2981 | 0.03 | 0.979 | 0.979 |
| Caudate nucleus | -0.28 | 2.81 | 2978 | -0.10 | 0.921 | 0.979 |
| Putamen | 3.52 | 3.66 | 2935 | 0.96 | 0.336 | 0.979 |
| Pallidum | 0.44 | 1.51 | 2879 | 0.29 | 0.771 | 0.979 |
| Hippocampus | -0.45 | 2.37 | 2973 | -0.19 | 0.849 | 0.979 |
| Amygdala | 1.71 | 1.18 | 2963 | 1.45 | 0.148 | 0.979 |
| Nucleus accumbens | -0.03 | 0.60 | 2951 | -0.06 | 0.954 | 0.979 |

**Table S10** Significance of the nonlinearity of BMI effects (BMI quartile by within-quartile BMI interaction) in predicting cortical thickness. FDR-adjusted *p*-values are shown.

| *Region* | *Nonlinear BMI significance* |
| --- | --- |
| Banks STS | χ2=0.77, DF=1, p=0.865 |
| Caudal anterior cingulate | χ2=0.43, DF=1, p=0.865 |
| Caudal middle frontal | χ2=0.56, DF=1, p=0.865 |
| Cuneus | χ2=0.14, DF=1, p=0.913 |
| Entorhinal | χ2=0.97, DF=1, p=0.823 |
| Fusiform | χ2=0.36, DF=1, p=0.869 |
| Inferior parietal | χ2=0.01, DF=1, p=0.985 |
| Inferior temporal | χ2=0.37, DF=1, p=0.869 |
| Isthmus cingulate | χ2=1.66, DF=1, p=0.735 |
| Lateral occipital | χ2=0.15, DF=1, p=0.913 |
| Lateral orbitofrontal | χ2=0.05, DF=1, p=0.936 |
| Lingual | χ2=0.97, DF=1, p=0.823 |
| Medial orbitofrontal | χ2=0.08, DF=1, p=0.936 |
| Middle temporal | χ2=0.01, DF=1, p=0.997 |
| Parahippocampal | χ2=0.53, DF=1, p=0.865 |
| Paracentral | χ2=0.06, DF=1, p=0.936 |
| Pars opercularis | χ2=0.27, DF=1, p=0.878 |
| Pars orbitalis | χ2=0.74, DF=1, p=0.865 |
| Pars triangularis | χ2=0.68, DF=1, p=0.865 |
| Pericalcarine | χ2=0.02, DF=1, p=0.985 |
| Postcentral | χ2=0.28, DF=1, p=0.878 |
| Posterior cingulate | χ2=1.85, DF=1, p=0.735 |
| Precentral | χ2=0.01, DF=1, p=0.985 |
| Precuneus | χ2=1.62, DF=1, p=0.735 |
| Rostral anterior cingulate | χ2=0.18, DF=1, p=0.903 |
| Rostral middle frontal | χ2=0.24, DF=1, p=0.892 |
| Superior frontal | χ2=0.10, DF=1, p=0.932 |
| Superior parietal | χ2=0.71, DF=1, p=0.865 |
| Superior temporal | χ2=0.01, DF=1, p=0.985 |
| Supramarginal | χ2=0.08, DF=1, p=0.936 |
| Frontal temporal | χ2=0.29, DF=1, p=0.878 |
| Temporal pole | χ2=0.48, DF=1, p=0.865 |
| Transverse temporal | χ2=0.64, DF=1, p=0.865 |
| Insula | χ2=0.13, DF=1, p=0.913 |

**Table S11** Significance of the nonlinearity of BMI effects (BMI quartile by within-quartile BMI interaction) in predicting cortical surface area. FDR-adjusted *p*-values are shown.

| *Region* | *Nonlinear BMI significance* |
| --- | --- |
| Banks STS | χ2=0.88, DF=1, p=0.824 |
| Caudal anterior cingulate | χ2=0.45, DF=1, p=0.865 |
| Caudal middle frontal | χ2=1.10, DF=1, p=0.823 |
| Cuneus | χ2=0.01, DF=1, p=0.985 |
| Entorhinal | χ2=4.57, DF=1, p=0.279 |
| Fusiform | χ2=5.20, DF=1, p=0.250 |
| Inferior parietal | χ2=1.48, DF=1, p=0.774 |
| Inferior temporal | χ2=4.58, DF=1, p=0.279 |
| Isthmus cingulate | χ2=5.53, DF=1, p=0.250 |
| Lateral occipital | χ2=0.05, DF=1, p=0.936 |
| Lateral orbitofrontal | χ2=1.08, DF=1, p=0.823 |
| Lingual | χ2=2.31, DF=1, p=0.654 |
| Medial orbitofrontal | χ2=0.21, DF=1, p=0.903 |
| Middle temporal | χ2=5.59, DF=1, p=0.250 |
| Parahippocampal | χ2=9.64, DF=1, p=0.152 |
| Paracentral | χ2=5.70, DF=1, p=0.250 |
| Pars opercularis | χ2=0.01, DF=1, p=0.985 |
| Pars orbitalis | χ2=0.52, DF=1, p=0.865 |
| Pars triangularis | χ2=0.17, DF=1, p=0.903 |
| Pericalcarine | χ2=1.94, DF=1, p=0.735 |
| Postcentral | χ2=1.34, DF=1, p=0.801 |
| Posterior cingulate | χ2=1.31, DF=1, p=0.801 |
| Precentral | χ2=3.73, DF=1, p=0.316 |
| Precuneus | χ2=2.34, DF=1, p=0.654 |
| Rostral anterior cingulate | χ2=5.31, DF=1, p=0.250 |
| Rostral middle frontal | χ2=0.28, DF=1, p=0.878 |
| Superior frontal | χ2=0.92, DF=1, p=0.824 |
| Superior parietal | χ2=5.52, DF=1, p=0.250 |
| Superior temporal | χ2=0.01, DF=1, p=0.985 |
| Supramarginal | χ2=0.97, DF=1, p=0.823 |
| Frontal temporal | χ2=1.69, DF=1, p=0.735 |
| Temporal pole | χ2=0.17, DF=1, p=0.903 |
| Transverse temporal | χ2=0.36, DF=1, p=0.869 |
| Insula | χ2=3.80, DF=1, p=0.316 |

**Table S12** Significance of the nonlinearity of BMI effects (BMI quartile by within-quartile BMI interaction) in predicting cortical thickness. FDR-adjusted *p*-values are shown.

| *Region* | *Nonlinear BMI significance* |
| --- | --- |
| Ventricles | χ2=0.99, DF=1, p=0.823 |
| Thalamus | χ2=0.48, DF=1, p=0.865 |
| Caudate | χ2=4.35, DF=1, p=0.281 |
| Putamen | χ2=4.05, DF=1, p=0.304 |
| Pallidum | χ2=0.51, DF=1, p=0.865 |
| Hippocampus | χ2=0.01, DF=1, p=0.985 |
| Amygdala | χ2=0.05, DF=1, p=0.936 |
| Accumbens | χ2=1.82, DF=1, p=0.735 |

**Table S13** Among participants with schizophrenia taking atypical antipsychotics at the time of scanning, the effects of BMI on cortical thickness when modeled alone, and the partial effects of BMI and antipsychotic medication dose on cortical thickness (chlorpromazine equivalent, in mg). Effect sizes (part r) are shown with FDR-adjusted *p*-values.

|  |  | BMI | | BMI partial | | Chlorpromazine eq. | |
| --- | --- | --- | --- | --- | --- | --- | --- |
| *Region* | *n* | *r* | *p* | *r* | *p* | *r* | *p* |
| Banks STS | 701 | -0.112 | 0.014 | -0.114 | 0.011 | -0.082 | 0.234 |
| Caudal anterior cingulate | 726 | -0.004 | 0.918 | -0.005 | 0.899 | -0.033 | 0.465 |
| Caudal middle frontal | 731 | -0.110 | 0.014 | -0.112 | 0.011 | -0.046 | 0.341 |
| Cuneus | 732 | -0.048 | 0.267 | -0.049 | 0.257 | -0.036 | 0.445 |
| Entorhinal | 727 | -0.062 | 0.152 | -0.064 | 0.140 | -0.073 | 0.234 |
| Fusiform | 731 | -0.065 | 0.139 | -0.066 | 0.128 | -0.032 | 0.465 |
| Inferior parietal | 728 | -0.097 | 0.029 | -0.098 | 0.026 | -0.047 | 0.341 |
| Inferior temporal | 721 | -0.005 | 0.916 | -0.007 | 0.869 | -0.087 | 0.234 |
| Isthmus cingulate | 735 | -0.020 | 0.651 | -0.021 | 0.632 | -0.039 | 0.425 |
| Lateral occipital | 733 | -0.123 | 0.014 | -0.124 | 0.011 | -0.039 | 0.425 |
| Lateral orbitofrontal | 734 | -0.058 | 0.178 | -0.059 | 0.171 | -0.030 | 0.480 |
| Lingual | 733 | -0.037 | 0.384 | -0.039 | 0.365 | -0.069 | 0.235 |
| Medial orbitofrontal | 732 | -0.075 | 0.095 | -0.075 | 0.096 | 0.005 | 0.902 |
| Middle temporal | 722 | -0.066 | 0.139 | -0.069 | 0.116 | -0.074 | 0.234 |
| Parahippocampal | 737 | -0.016 | 0.715 | -0.017 | 0.690 | -0.058 | 0.270 |
| Paracentral | 732 | -0.108 | 0.014 | -0.110 | 0.011 | -0.085 | 0.234 |
| Pars opercularis | 737 | -0.043 | 0.326 | -0.045 | 0.302 | -0.060 | 0.270 |
| Pars orbitalis | 733 | -0.080 | 0.076 | -0.080 | 0.074 | -0.011 | 0.792 |
| Pars triangularis | 736 | -0.086 | 0.060 | -0.087 | 0.056 | -0.036 | 0.445 |
| Pericalcarine | 712 | -0.031 | 0.461 | -0.032 | 0.457 | -0.011 | 0.792 |
| Postcentral | 689 | -0.073 | 0.112 | -0.075 | 0.102 | -0.062 | 0.270 |
| Posterior cingulate | 734 | -0.034 | 0.422 | -0.037 | 0.382 | -0.071 | 0.234 |
| Precentral | 692 | -0.120 | 0.014 | -0.123 | 0.011 | -0.094 | 0.234 |
| Precuneus | 731 | -0.109 | 0.014 | -0.112 | 0.011 | -0.078 | 0.234 |
| Rostral anterior cingulate | 728 | -0.119 | 0.014 | -0.121 | 0.011 | -0.046 | 0.341 |
| Rostral middle frontal | 731 | -0.078 | 0.084 | -0.079 | 0.077 | -0.052 | 0.325 |
| Superior frontal | 733 | -0.097 | 0.029 | -0.098 | 0.026 | -0.056 | 0.289 |
| Superior parietal | 730 | -0.114 | 0.014 | -0.116 | 0.011 | -0.058 | 0.270 |
| Superior temporal | 721 | -0.083 | 0.073 | -0.086 | 0.058 | -0.066 | 0.259 |
| Supramarginal | 725 | -0.069 | 0.124 | -0.070 | 0.115 | -0.049 | 0.341 |
| Frontal temporal | 735 | -0.049 | 0.267 | -0.049 | 0.257 | -0.032 | 0.465 |
| Temporal pole | 734 | -0.061 | 0.152 | -0.062 | 0.150 | -0.018 | 0.687 |
| Transverse temporal | 738 | -0.120 | 0.014 | -0.122 | 0.011 | -0.064 | 0.259 |
| Insula | 729 | -0.038 | 0.384 | -0.039 | 0.365 | -0.050 | 0.341 |

**Table S14** Among participants with schizophrenia taking atypical antipsychotics at the time of scanning, the effects of BMI on cortical surface area when modeled alone, and the partial effects of BMI and antipsychotic medication dose on cortical surface area (chlorpromazine equivalent, in mg). Effect sizes (part r) are shown with FDR-adjusted *p*-values.

|  |  | BMI | | BMI partial | | Chlorpromazine eq. | |
| --- | --- | --- | --- | --- | --- | --- | --- |
| *Region* | *n* | *r* | *p* | *r* | *p* | *r* | *p* |
| Banks STS | 693 | 0.060 | 0.343 | 0.060 | 0.350 | -0.009 | 0.951 |
| Caudal anterior cingulate | 728 | 0.086 | 0.185 | 0.086 | 0.185 | 0.004 | 0.951 |
| Caudal middle frontal | 733 | 0.068 | 0.343 | 0.068 | 0.350 | 0.028 | 0.940 |
| Cuneus | 729 | 0.008 | 0.914 | 0.009 | 0.898 | 0.025 | 0.940 |
| Entorhinal | 724 | -0.027 | 0.775 | -0.026 | 0.779 | 0.018 | 0.940 |
| Fusiform | 733 | 0.067 | 0.343 | 0.067 | 0.350 | 0.013 | 0.951 |
| Inferior parietal | 727 | 0.018 | 0.786 | 0.019 | 0.779 | 0.017 | 0.940 |
| Inferior temporal | 723 | 0.002 | 0.968 | 0.000 | 0.999 | -0.073 | 0.601 |
| Isthmus cingulate | 734 | 0.052 | 0.343 | 0.054 | 0.350 | 0.080 | 0.523 |
| Lateral occipital | 729 | 0.089 | 0.185 | 0.088 | 0.185 | -0.021 | 0.940 |
| Lateral orbitofrontal | 734 | 0.078 | 0.246 | 0.077 | 0.255 | -0.030 | 0.940 |
| Lingual | 735 | 0.054 | 0.343 | 0.053 | 0.350 | -0.030 | 0.940 |
| Medial orbitofrontal | 732 | 0.052 | 0.343 | 0.051 | 0.360 | -0.055 | 0.940 |
| Middle temporal | 724 | 0.027 | 0.775 | 0.027 | 0.779 | -0.016 | 0.940 |
| Parahippocampal | 729 | 0.006 | 0.937 | 0.006 | 0.930 | 0.018 | 0.940 |
| Paracentral | 726 | 0.057 | 0.343 | 0.055 | 0.350 | -0.085 | 0.523 |
| Pars opercularis | 729 | 0.025 | 0.779 | 0.024 | 0.779 | -0.038 | 0.940 |
| Pars orbitalis | 729 | 0.062 | 0.343 | 0.062 | 0.350 | 0.006 | 0.951 |
| Pars triangularis | 724 | 0.058 | 0.343 | 0.058 | 0.350 | -0.004 | 0.951 |
| Pericalcarine | 710 | -0.014 | 0.824 | -0.015 | 0.817 | -0.025 | 0.940 |
| Postcentral | 693 | -0.002 | 0.968 | -0.002 | 0.985 | -0.031 | 0.940 |
| Posterior cingulate | 733 | 0.062 | 0.343 | 0.062 | 0.350 | -0.009 | 0.951 |
| Precentral | 688 | 0.039 | 0.581 | 0.039 | 0.593 | -0.033 | 0.940 |
| Precuneus | 732 | 0.020 | 0.782 | 0.019 | 0.779 | -0.020 | 0.940 |
| Rostral anterior cingulate | 734 | 0.093 | 0.185 | 0.092 | 0.185 | -0.046 | 0.940 |
| Rostral middle frontal | 729 | 0.088 | 0.185 | 0.088 | 0.185 | 0.001 | 0.976 |
| Superior frontal | 732 | 0.049 | 0.385 | 0.048 | 0.404 | -0.053 | 0.940 |
| Superior parietal | 729 | 0.053 | 0.343 | 0.053 | 0.350 | 0.012 | 0.951 |
| Superior temporal | 718 | 0.013 | 0.825 | 0.013 | 0.824 | 0.004 | 0.951 |
| Supramarginal | 727 | 0.021 | 0.782 | 0.020 | 0.779 | -0.036 | 0.940 |
| Frontal temporal | 738 | 0.037 | 0.581 | 0.036 | 0.593 | -0.023 | 0.940 |
| Temporal pole | 736 | -0.021 | 0.782 | -0.021 | 0.779 | 0.007 | 0.951 |
| Transverse temporal | 734 | 0.017 | 0.792 | 0.016 | 0.799 | -0.018 | 0.940 |
| Insula | 726 | 0.020 | 0.782 | 0.020 | 0.779 | 0.026 | 0.940 |

**Table S15** Among participants with schizophrenia taking atypical antipsychotics at the time of scanning, the effects of BMI on subcortical volume when modeled alone, and the partial effects of BMI and antipsychotic medication dose on subcortical volume (chlorpromazine equivalent, in mg). Effect sizes (part r) are shown with FDR-adjusted *p*-values. Significance is shown with asterisks (*, α=0.05).

|  |  | BMI | | BMI partial | | Chlorpromazine eq. | |
| --- | --- | --- | --- | --- | --- | --- | --- |
| *Region* | *n* | *r* | *p* | *r* | *p* | *r* | *p* |
| Ventricles | 711 | -0.015 | 0.699 | -0.012 | 0.745 | 0.056 | 0.534 |
| Thalamus | 731 | 0.090 | 0.064 | 0.089 | 0.067 | -0.045 | 0.534 |
| Caudate | 734 | 0.039 | 0.579 | 0.040 | 0.558 | 0.038 | 0.534 |
| Putamen | 729 | 0.031 | 0.650 | 0.032 | 0.635 | 0.027 | 0.534 |
| Pallidum | 723 | 0.016 | 0.699 | 0.016 | 0.745 | 0.029 | 0.534 |
| Hippocampus | 734 | 0.046 | 0.579 | 0.044 | 0.558 | -0.073 | 0.406 |
| Amygdala | 732 | 0.116 | 0.015 | 0.116 | 0.015 | -0.008 | 0.834 |
| Accumbens | 728 | 0.023 | 0.699 | 0.024 | 0.687 | 0.035 | 0.534 |

**Figure S1:** BMI distribution of all participants


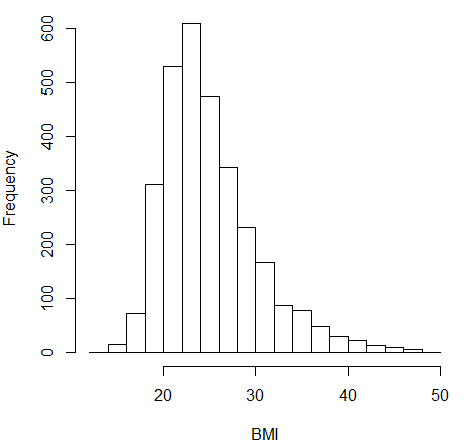


**Linear vs. nonlinear BMI influence on outcome measures**

An important question was whether the influence of BMI on regional measures was linear or nonlinear. To investigate this, we categorized individuals within BMI categories: 1) 15-19.9, 2) 20-24.9, 3) 25-29.9, or 4) 30-34.9. Participants were also scored according to their relative BMI within categories, ranging from +0 to +4.9. A nonlinear association between BMI and an outcome measure would manifest as differences between BMI categories in terms of the regression slope for within-category predictions. This was tested using an interaction between BMI category and relative BMI within categories for each region. The results for these interactions are shown in Table S10, S11 and S12. This interaction for a representative region is shown in Figure S2 (Cortical thickness of rostral anterior cingulate gyrus), where each incremental BMI category is associated with lower cortical thickness, but the slope within each category is similar due to the non-significant interaction, suggesting a linear effect of BMI in this region.


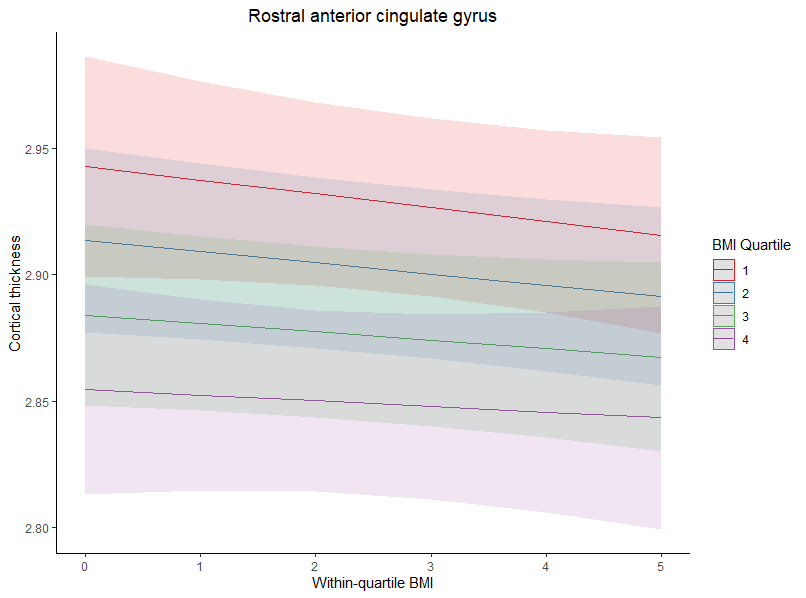


**Figure S2** The interaction between BMI categories and within-category relative BMI in predicting rostral anterior cingulate gyrus thickness, demonstrating equivalent slopes at all BMI ranges, for an approximately linear relationship.

***Supplemental Methods***

***Description of fixed and random effects in each model***

Model specifications for each analysis type are outlined below, in *R* syntax for regression models. In each case, *Dx* refers to the categorical grouping of participants (participant with schizophrenia or healthy control), and *ICV* (total intracranial volume in mm^3^) was used to control for overall volume differences between individuals. In the analysis of medication effects, *Dose* refers to the prescribed antipsychotic dosage in chlorpromazine equivalent (mg), and models investigating (4) dosage effects only included participants with schizophrenia. *Hemisphere* (left or right) controlled for inter-hemisphere volume differences for a region, both overall (fixed effect) and within individuals (random effect), allowing for estimation of a region bilaterally using a single model, with one model per region per outcome measure (cortical thickness, cortical surface area, or subcortical volume). In addition to the fixed effects specified below, every model used the same random effect structure (1 + hemisphere | Site / Subject), allowing for random variability both between individuals and between sites overall.

1. *Outcome† = Dx + BMI + Age + Sex + hemisphere + ICV* *‡*
2. *Outcome† = BMI + Dose + Age + Sex + hemisphere + ICV* *‡*

*† Outcome was either cortical thickness (mm), cortical surface area (mm^2^), or subcortical volume (mm^3^)
‡ ICV was included as a covariate when outcome was either cortical surface area or subcortical volume*
